# How language, culture, and geography shape online dialogue: Insights from Koo

**DOI:** 10.1371/journal.pone.0329838

**Published:** 2025-08-21

**Authors:** Amin Mekacher, Max Falkenberg, Andrea Baronchelli

**Affiliations:** 1 Department of Mathematics, City St George’s, London, United Kingdom; 2 Department of Network and Data Science, Central European University, Vienna, Austria; University of Science and Technology of China, CHINA

## Abstract

Founded in India in 2020, the microblogging site ‘Koo’ launched as an alternative to mainstream social media platforms, with the explicit aim of catering to non-Western communities in their vernacular languages, and capitalising on a period of tension between the Indian government and Twitter which led many users to seek Twitter-alternatives. Drawing on a near-complete dataset totalling over 71M posts and 399M user interactions, we show how Koo attracted users from several countries including India, Nigeria and Brazil, but with variable levels of sustained user engagement. We highlight how Koo’s interaction network was shaped by multiple country-specific migrations displaying strong divides between linguistic and cultural communities, for instance, with English-speaking communities from India and Nigeria largely isolated from one another. Finally, we analyse the content shared by different linguistic communities and identify cultural patterns which, we speculate, promoted similar discourses across language groups. Our results show that for language groups of similar sizes, Indian languages fostered higher discourse diversity than non-Indian languages, possibly highlighting synergistic effects which boosted the uptake and retention of these groups. Despite this, Koo failed to capitalise on this synergy and ceased operations in July 2024. With this context, our study points to some of the possible reasons why the multilingual and politically diverse platform Koo struggled to remain sustainable, failing to stave off competition from its US-based competitors, despite its commitment to cultivating support for the different vernacular communities of Indian social media users.

## Introduction

The social media ecosystem, which has historically been Western-focused [1], has evolved significantly in recent years, with a rapidly growing number of active users from non-Western countries and the Global South [2]. Despite this shift in demographics, major social media platforms such as Twitter (now X) and Facebook lack full support for many major vernacular languages [3] (e.g., in South-East Asia [4]), with platforms continuing to prioritise Western audiences. For instance, investments into English-language content moderation on Twitter, Facebook and Instagram still heavily outstrip investments into moderation tools for other languages [5], indicating that platforms are less equipped to tackle, and are (arguably) less concerned about, harmful content posted in languages other than English. This is despite some evidence that social media can play a key role in election campaigns [6], and the fact that India, the World’s largest democracy, has become the largest market in terms of users for many leading social platforms including Instagram [7], Youtube [8] and Facebook [9].

According to its founders and management, addressing the persistent failure of social media platforms to support many communities beyond the West was the *raison d’etre* of Koo, a microblogging platform launched in Bangalore, India, in early 2020. Koo aimed to champion a “language-first” approach, where each user was able to express themselves in their native language when connecting with their peers [10]. By upscaling their auto-translate tool, Koo aimed to offer an inclusive experience to speakers of less widely utilized languages, a feature not prioritised on the dominant US-based platforms. Indicative of this, Koo supported 20 of India’s 22 official languages, whereas Twitter (now X) only supports 5 [11]. Importantly, however, languages other than those which are officially supported can be used on Twitter (and other platforms) even if they are not explicitly supported by the platform (for instance, Punjabi was used extensively on Twitter during the 2020 Farmer’s protests in India, despite being an unsupported language). By appealing to political leaders across countries, mostly from India, Nigeria and Brazil, including some who criticised US-based social media platforms, Koo managed to attract a geographically diverse user-base, becoming, for a period of time, the second largest microblogging platform globally after Twitter [12,13]. As such, it briefly occupied an influential position in the social media ecosystem, offering a unique opportunity to study the role of language on social media and offering insights as to the factors driving the sustainability of emerging social media platforms.

This paper follows our recent release of the Koo dataset [14], and an accompanying paper (published prior to the platform’s shutdown) in which we discussed the platform’s sustainability (relative to other platforms), its use in political debates, and how news is shared on the platform [15]. Although often defined as an alt-tech platform, Koo attracted a more international user base than US-based alt-tech platforms [16–18] leading to a more diverse community. Here, we extend this work by focusing explicitly on the role that language played in shaping the structure of a non-Western social platform.

Previous social media studies have considered the role of language in shaping online interactions, but not in the context of India. Researchers have studied linguistic trends on Twitter and found that English-speaking posts were dominant on the platform when it mostly attracted users from Western nations [19,20], whereas the usage of English became less prevalent when considering non-Western countries [21,22]. Moreover, following the growth of communities outside English-speaking nations, different linguistic communities were shown to interact differently with a social platform’s features, leading to distinct social structures [23]. However, despite the subsequent globalization of social media, the formation of dyadic ties on Twitter was found to be strongly correlated with the linguistic background of a user, even between different English-speaking countries [24,25]. Studies have also considered the influence of bilingual social media users on interaction networks, and whether users post in languages other than a platforms dominant language [26,27]. Language diversity was further quantified by using geolocation data to map the language diversity in the Greater Manchester area [28]. More recently, studies have compared literacy levels across regions on Facebook [29]. Our research contributes to this literature by studying an online platform striving to host vernacular languages, in the hope of harboring a sustainable multi-lingual community.

With 22 nationally-recognized languages, and over 100 languages with more than 10,000 native speakers, India is a unique case study to assess the impact of linguistic pluralism on user-to-user interactions online. India’s linguistic history has been the focus of several studies, looking into the national linguistic landscape [30], the patterns of communication [31] and the socio-economic ramifications of a complex linguistic environment [32], but many of these methods have not been applied in the context of social media. Moreover, the rich linguistic composition of India also allows for an analysis of language use at a national scale, before looking at linguistic communities across several countries. Similar observations are valid for Nigeria, one of the other countries where Koo was adopted by government officials, where multilingualism plays a major role in social interactions in several areas [33], with about 500 vernacular languages spoken across the country [34].

In the remainder of this paper we first assess the impact of the various collective migrations to Koo which shaped the platform into a multilingual venue. We then study how political incentives to migrate to Koo led to different degrees of user engagement over time. Afterwards, we look at the topology of the interaction network, while assessing the impact of the language barrier to foster cross-cultural communities. Finally, we look at user mobility across linguistic landscapes and how this relates to the richness of a community’s online conversation, as well as the shared discourse between language pairs. Our findings suggest that linguistic and cultural factors are instrumental in bridging communities on Koo, with few interactions taking place across communities with different linguistic backgrounds.

## Results

### Platform migration and user retention

**Fig 1:**
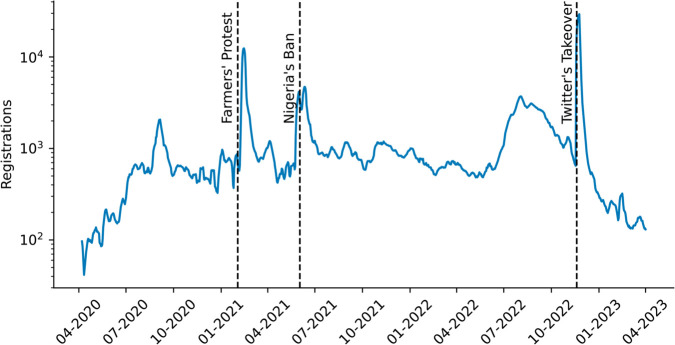
Daily number of registrations on Koo, and the impact of collective migration. 7-day moving average of the daily number of registrations on Koo, from the beginning of 2020 to early 2023. The dashed lines indicate, in order: the migration of BJP politicians and their supporters following the Indian Farmers’ Protest in February 2021; the migration of the Nigerian government after Twitter was banned in the country in June 2021; the Brazilian community joining Koo in November 2022 after Elon Musk purchased Twitter.

We begin by examining Koo’s popularity over time in the online ecosystem. Fig 1 shows the daily number of registrations on Koo, from the launch of the platform in 2020 until early 2023, the period for which our published dataset is near complete. Major political and social events, which had an impact on Koo’s outreach, are marked as dashed lines.

The first significant peak in registrations can be seen in February 2021. India was in the midst of the farmers’ protest, a popular movement against a new set of laws adopted by the Parliament of India in September 2020. These events triggered a conflict between the Indian government and Twitter after officials from the BJP (the ruling political party) pressured Twitter to ban accounts linked to the popular movement [35]. Members of the government and BJP supporters in India subsequently signed up to Koo, as Twitter did not comply with their requests, and invited their community to follow suit [36]. As seen on the figure, the political movement managed to increase Koo’s user base substantially [37]. The platform’s willingness to comply with content take down orders issued by the government made Koo more attractive to BJP politicians, thereby cementing the dominance of BJP narratives on the platform [36].

The second burst in registrations dates from June 2021, when Nigerian then-President Muhammadu Buhari banned Twitter from the country and registered on Koo with members of his government [38], after Buhari’s tweets were deleted for inciting violence against his political opponents [39]. Koo experienced a wide adoption from government officials in Nigeria, prompting the platform to hire vernacular speakers for content moderation purposes in Nigeria [40]. The government was also followed by a large number of Nigerian users who subsequently signed up to Koo, leading to an uptick in registrations. However, Koo had little success in attracting Nigerian celebrities or influencers, unlike in India where the platform gained support from Bollywood actors and prominent cricket players [41]. This is important given that previous research has shown that celebrities’ endorsement can catalyze large migrations towards alt-tech platforms [42].

The last major peak in registrations took place in November 2022, shortly after Twitter was purchased by Elon Musk. Felipe Neto, a Brazilian influencer with over 16 million followers on Twitter, advertised his migration to Koo on Twitter, which led to his followers signing up to the platform as well [43]. This collective movement was strengthened when Brazilian President Lula also registered on Koo [44]. In total, Koo’s user base grew substantially in Brazil, with the Koo app downloaded over 1 million times in the space of 48 hours [45].

These three events shaped the major linguistic communities on Koo. A breakdown of the registration numbers broken down by language is provided in the SI, showcasing the influx of Hindi, Nigerian English, and Portuguese speaking users.

To assess the success of linguistic migrations, we measure user retention, i.e. how many users within a cohort are still active after a given number of days. Throughout our analysis, we match each user with the language they used the most when posting and commenting on the platform.

**Fig 2:**
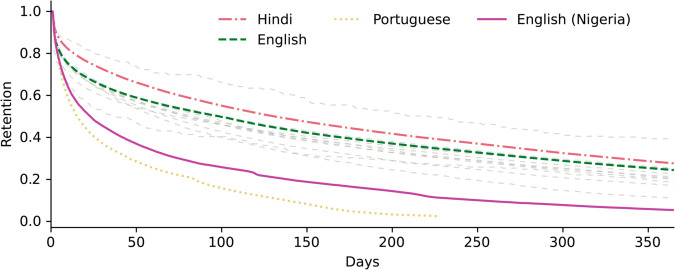
Heterogeneous user retention for various linguistic communities. Kaplan-Meier survival curves for the main linguistic communities on Koo, showing the fraction of users who remained active after a given number of days. For each user, we define “day zero” as being their registration date on Koo. The largest linguistic communities active on Koo are shown in color (Hindi: pink, dash-dot. English: green, dashed. Portuguese: yellow, dotted. English (Nigeria): purple, solid.). Other linguistic communities are displayed in gray. The retention curve is displayed until the day that fewer than 1% of users from a linguistic community remain active.

Fig 2 shows the Kaplan-Meier estimator, a tool used to visualize the retention curve of a population over time, computed for each linguistic community on Koo. Given a linguistic community, the Kaplan-Meier estimator indicates how many users were still active on Koo, a given number of days after they registered on the platform. The figure indicates that both Brazilian and Nigerian communities had lower retention than other linguistic communities on Koo, with 50% of the cohort becoming inactive within 16 days and 23 days of signing up to the platform, respectively. In contrast, it took 131 days to reach the same level of user retention when considering Hindi-speaking users, thus highlighting a strong difference in user engagement across linguistic communities and countries. The smaller linguistic clusters also display a higher survival rate than the Brazilian and Nigerian users, which suggest that the sustainability of a community does not only depend on its population size.

The lack of long-term adoption in the Nigerian community can be explained by a popular resistance to the Twitter ban that was instated by Buhari’s government in June 2021. Nigerian users managed to bypass the ban shortly after it was instated, with VPN usage becoming more common nation-wide [38]. Moreover, Koo did not receive sustained support from the Nigerian government. Muhammadu Buhari lifted the ban on Twitter in January 2022, after the platform and his government settled on an agreement [46]. Buhari stopped being active on Koo shortly afterwards, as did most of the government members who joined the platform. Koo’s monthly active users in Nigeria fell by over 90%, suggesting that the platform failed to establish a foothold as sustainable as their popularity in India [47].

In the case of Brazil, the migration was not, primarily, triggered by political motivations in the same way as for India and Nigeria, but rather by a linguistic pun involving the word “koo” in Portuguese, although Brazilian President Lula did join the platform during its initial growth-phase. However, in general, the Brazilian community and Brazilian celebrities did not stay as engaged on Koo, when compared to celebrities from India [48]. Both Felipe Neto and Lula, who were the main drivers of the Brazilian migration to Koo, stayed active on Twitter and their followers were therefore still able to follow their feed without requiring access to Koo.

Previous research has shown that users are less active on alt-tech platforms if they can reach their followers via a mainstream outlet [42]. However, across the time period covered by our dataset, the retention of Hindi-speaking users on Koo remained higher than for other language groups, despite many Hindi-speaking celebrities, influencers, and politicians remaining active on Twitter. The reasons for this discrepancy are not fully clear, but may be explained by noting that throughout 2021, the Indian government expressed frequent and concerted criticism of Twitter [36], especially its refusal to remove content related to the Farmer’s protest, and this may have resulted in extended political support for the Koo platform across many months, not just across a short time period as was the case in previous studies [42]. We note that tensions between Twitter and the Indian government eased after 2021, possibly contributing to Koo’s struggle to remain a viable competitor to Twitter, but that new tensions concerning content moderation have emerged in 2025 following the platform’s takeover by Elon Musk and Twitter’s rebranding to X.

These results suggest that collective migrations to an alternative social media platform can have mixed levels of success, depending on the motivations triggering the migration and the degree of approval it garners across the community. The Indian migration exemplifies the birth of, what initially appeared to be, a sustainable community on Koo, as it was led by government officials and garnered support from both national celebrities and BJP supporters. The Nigerian government followed a different pattern, where the low support for the Twitter ban outside of Buhari’s supporters led to a short-lived retention for most Nigerian users who signed up to Koo. In the same fashion, the Brazilian community showed low user retention, which can be explained by a lack of social incentives to shift the political discourse to an alternative platform, and away from the dominant US-based platforms.

### Language-use in the Koo interaction network

We now focus on user interactions on Koo and the landscape that emerges on a platform where many languages and cultures coexist, following individual migration decisions.

**Fig 3:**
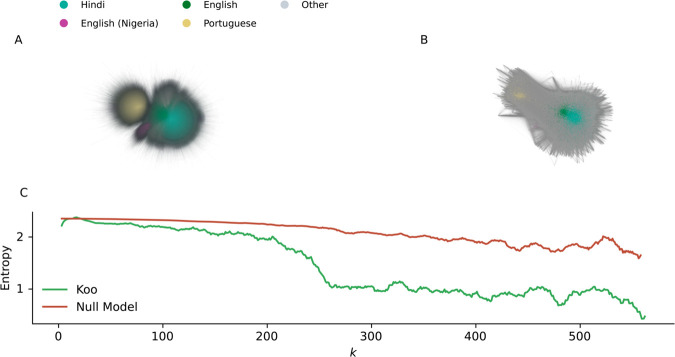
The Koo interaction network and the impact of linguistic homophily on the network’s structure. Each node represents a user, and two nodes are connected if one of the users interacted with the other user’s content. Users are colored according to their modal language on the platform. The main linguistic communities are the Hindi-speaking users (blue), English-speaking users (green), Nigerian users (purple) and Portuguese-speaking users (yellow). The layout is generated by using a force-directed graph drawing algorithm. A) The total interaction network. B) The *k*-core of the interaction network with *k* = 150. C) The Shannon entropy of the modal language of the nodes belonging to the k-core of the graph, with respect to the value of *k*. The entropy of the interaction network is compared to the value obtained in a null model, where we shuffle the modal language associated to each node in the network. For an alternative network visualization with greater clarity of the different language groups active on Koo, we refer the reader to Fig 2 in our original Koo dataset paper [15] where we present a network of “accounts of eminence” on Koo (similar to verified users) labeled according to language.

Fig 3A shows an interaction network, where two users are connected if they interacted (i.e. one of the users liked, shared or commented the other’s post) on Koo, with the weight of an edge proportional to the number of interactions between a pair of users; for simplicity we treat the interaction network as undirected. The network layout, generated with a force-directed graph drawing algorithm, highlights the strong segregation between several linguistic communities: Portuguese-speaking users (yellow) mostly interact with other Portuguese-speaking users, and likewise, Hindi-speaking users (blue) mostly interact with other Hindi-speaking users. On the other hand, the English-speaking cohort (green) acts as a bridge between Hindi, Portuguese and Nigerian English speakers (purple), as well as the smaller cohorts that can be seen in the periphery of the graph.

The network suggests a strong homophilic behavior, meaning that users will mainly engage with members of their own linguistic community. Koo’s auto-translate feature, which allows users to translate any post to the language of their choice, does not seem to have mitigated the language assortativity that we observe on the platform. Homophilic patterns have been observed in many online social spaces, for example when considering information diffusion dynamics [49], community formation [50], and in political interaction networks [25]. Unfortunately, robust quantitative data on language assortativity is rare, especially given that most social media studies focus on a single language and use sampled data (which can potentially have major impacts on any subsequent analysis [51]), but one study suggests that assortativity by language is 0.56 for follower networks on Twitter and 0.74 for retweet networks, in contrast to assortativity by degree which is negative (disassortative) in both cases [52].

We can highlight this homophilic behavior by computing the *k*-cores of the network. Given a network, its *k*-core for k∈ℕ is defined as the sub-graph in which all vertices have a degree greater than or equal to *k*. *k*-core analysis offers a deeper view into the tightly connected components of a network, therefore identifying the nodes that are strongly interconnected or play an influential role in the overall network structure [53]. Fig 3B shows the interaction network from Fig 3A but filtered so that only the k≥150 core is shown. This threshold allows us to filter out the periphery of the network, by only keeping the 5% most strongly connected nodes of the network.

This visualization offers a better overview of the tightly interconnected components within the interaction network, with a strong core of English-speaking and Hindi-speaking users. Conversely, the Brazilian and Nigerian clusters are more isolated. The *k*-core also highlights that users belonging to smaller linguistic communities are rarely in the core of the interaction network, with 95% of the core users belonging to the Hindi, English, Nigerian English and Portuguese-speaking clusters. This analysis further underlines that highly connected clusters mostly involve users who speak one of the languages which were dominant on the platform. Looking at the English-speaking community, we note that it was principally connected to Hindi speakers in the *k*-core, highlighting the instrumental role that the English language plays in Indian political communications [54]. On the other hand, both the Nigerian and the Brazilian communities communicated primarily in their native language.

To quantify the connection between linguistic communities, we generate the *k*-core for all values of *k* for which the *k*-core exists. We retrieve the modal language of each vertex included in the *k*-core and compute the Shannon entropy, to evaluate whether the *k*-core encompasses a diverse range of languages or is primarily dominated by a few major languages [55,56]. Once we have computed the *k*-core, we retrieve the modal language of each user included in the core and calculate the entropy of the list of languages. Fig 3C displays the resulting entropy with respect to *k*. We notice that higher values of *k* lead to a lower entropy in the language composition of the *k*-core, suggesting that dense interactions on Koo take place mostly within homogeneous linguistic clusters.

To ensure that the lack of diversity in high-degree interactions is a characteristic of the interaction network on Koo, and not an erroneous finding, we define a null-model of the network, where the modal language is shuffled for the nodes in the network (preserving the language prevalence distribution), and the Shannon entropy is computed again for each value of *k*. Fig 3C displays the median Shannon entropy for each value of *k*, after running the null-model 1000 times. We notice that the entropy for the null-model does not sharply decrease for higher values of *k*, which reveals that this sharp decline in language diversity within the k-core is indeed a distinctive feature of the Koo interaction network. These findings further indicate that cross-linguistic interactions on Koo are rare with respect to same-language interactions.

To measure the prevalence of a linguistic community within the k-core for any value of *k*, vertices in the network can also be defined by their coreness, i.e., the maximum value of *k* for which they still belong to the *k*-core. In the SI, we show the distribution of the coreness of the users belonging to each linguistic community. Our analysis reveals that only Hindi- and English-speaking users are included in the highest cores. This result indicates that, despite the presence of a rich linguistic landscape on the platform, strong interaction ties on Koo are mainly driven by linguistic homophily, and that cross-linguistic interactions are rare.

### Language homophily and multilingual activity

The structure of the interaction network indicates that the linguistic background of a user strongly influenced their interaction patterns on Koo. Previous studies have highlighted that many ties in social networks are strongly assortative, i.e., they connect individuals who share similar attributes in terms of cultural background or social status [57]. Language assortativity has been found to influence mating decisions [58], friendship ties among adolescents [59], and political communication between countries using a common language [25].

When considering a diverse population such as the Koo user base, a question to consider is the interaction patterns for members of minority linguistic groups. Previous studies have shown that minority groups in organizations rely on out-group interactions to be connected to the center of the network [60], whereas belonging to an under-represented social group leads to a stronger in-group identity in friendship networks [61]. As such, we will next look at linguistic behaviors on Koo, by measuring the propensity for users to interact within their linguistic community on the platform. We will also evaluate a user’s likelihood of using their modal language when interacting with their peers on Koo.

To measure a user’s adherence to their modal language relative to their propensity to use other languages, we define language commitment: given a user with N posts in their modal languages and M posts in other languages, the commitment is given by

$$C=\frac{N}{N+M}.$$ (1)

Commitment has previously been used in linguistic studies to assess the adoption of new linguistic norms, indicating that outdated norms are still persistently used by a minority of the population [62]. Similar findings have been highlighted in ethnographic studies looking at the adoption of new spelling rules in both Spanish and English-speaking nations [63,64]. The literature therefore suggests the potential for smaller-scale communities to survive in a linguistic setup, despite the rise of a dominant linguistic framework.

**Fig 4:**
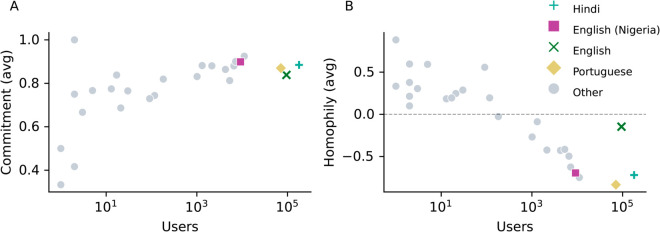
Language commitment and EI-Homophily relative to a language community’s size. Number of users belonging to a linguistic community plotted against A) their commitment to their modal language, and B) their EI homophily index. Both metrics are averaged by the number of users for whom the language measured is their modal language. The colored dots represent the Hindi-speaking community (blue), English (green), Portuguese (yellow) and Nigerian English (purple). The dashed line indicates an average homophily equal to 0.

The average commitment of a linguistic community, plotted against its population size, is displayed in Fig 4A. We notice that the average commitment of a user to their modal language increases as the population size of their community increases. Among the highlighted communities, Nigerian English speakers display the highest average commitment (*C* = 0.90), and English-speaking users also display a high level of commitment (*C* = 0.84), despite their role as a bridging community between other languages. Portuguese and Hindi speakers also have a high commitment (*C* = 0.87 and *C* = 0.88, respectively), which indicates that their communication on Koo mostly relied on their modal language, emphasizing the absence of cross-language interactions. However, smaller linguistic communities display a lower commitment to their modal language. For example, Indian communities, such as Odia speakers (*C* = 0.73), and non-Indian clusters such as Spanish speakers (*C* = 0.74), are less committed to their modal language. This finding highlights the need to communicate in other languages in order to be part of a community on the platform. The attractiveness of a language has previously been modeled by its number of speakers [65] suggesting that smaller linguistic communities are less likely to attract new speakers, thus fueling their need to use other languages in order to be connected to core conversations on the platform.

The high level of commitment observed for the major linguistic communities, along with the topology of the interaction network, shows that there is a strong trend for interactions on the platform to involve two users with a similar linguistic background. To measure whether social interactions on Koo mostly take place within the confines of homogeneous linguistic clusters, we use the External-Internal (EI) homophily index [66]: given a node in the interaction network with E edges to their out-group (in this case, interactions with another linguistic community) and I edges with their in-group (members of the same linguistic community), their EI homophily index is given by

$$EI=\frac{E-I}{E+I}.$$ (2)

A node which only interacts within their in-group (same language) therefore has *EI* = −1, whereas a node which only interacts with their out-group (different languages) has *EI* = 1. The EI-homophily index has previously been used to measure people’s tendency to interact with their politically-aligned peers on social media [67] and those sharing a similar vaccination status [68], with both studies showing an overall trend for people to cluster in homogeneous groups. A social network where the majority of interactions are intra-group links (i.e. with the EI-homophily index close to −1) is referred to as being *homophilic*, whereas it is referred to as *heterophilic* if the network displays several inter-group interactions (i.e. with an EI-homophily index close to 1). We note, however, that the exact value of the EI-homophily can be biased by unbalanced group sizes, so it is important to contextualize numerical results appropriately (see below).

The EI-homophily, averaged for each linguistic community by considering the modal language of each user, is shown in Fig 4B, and plotted against the size of each language’s population on Koo. We notice that small communities have a positive homophily index, indicating that members of small linguistic clusters interact mostly with other linguistic communities. On the other hand, with the exception of the English-speaking community, larger linguistic communities are more involved in in-group interactions, leading to a negative EI-homophily index. We note that this general trend is, partly, a known bias of the EI-homophily index; even if small language communities want to be homophilic in their interactions, the limited size of the community limits the maximum number of in-group interactions that a user can have, while permitting a large number of out-group interactions. For this reason, we expect small language communities to, on average, have larger EI-homophily than larger language communities. However, this bias also highlights that, even if Koo wanted to foster smaller language communities, in-group interactions are limited if a community is insufficiently large. Future work should consider repeating this analysis by applying a null model which corrects for community size.

For large language communities with similar population sizes, the EI-homophily of groups can be fairly compared. For example, Portuguese-speaking users have an average EI-homophily index of –0.94, in contrast to –0.66 for the Hindi-speaking users. This finding highlights the existence of siloed communities on Koo, where users’ interactions are strongly influenced by language similarities. The strong assortativity with respect to language is further displayed in the layout of the interaction network in Fig 3A, where we see how disjointed the Portuguese-speaking and Hindi-speaking communities are with respect to other linguistic communities.

Looking at the average EI-homophily index for English-speaking communities in Fig 4B, we notice a stark difference between Nigerian English speakers (*EI* = −0.64) and other English speakers (*EI* = −0.15). This can also been observed in the interaction network in Fig 3A, where Nigerian English-speaking users are disconnected from the core of the network, whereas English speakers are strongly involved in cross-language interactions, leading to a higher average EI-homophily index than for Hindi and Portuguese speakers. Thus, the English language acts as a *lingua franca* on Koo, allowing users from diverse linguistic backgrounds to engage in inter-community interactions. However, the contrast in homophily between Nigerian English and English speakers also highlights that language is not the only factor playing a central role in shaping the interaction network. Cultural similarities are also influential in bridging users together on a social platform. Communities can be structured around salient topics of conversation related to the cultural and political landscape of a country, another feature of a platform hosting diverse demographics that we investigate below. Moreover, it is likely that most of the English speakers which were active on Koo (excluding those who speak Nigerian English) had an Indian focus, due to the ubiquity of the English language in India’s public affairs [69].

The homophilic patterns observed in the interaction network suggest that communication across linguistic communities was rare on Koo. However, our analysis does not take into account users who communicate on the platform in more than one language, and therefore belong to more than one linguistic community on the platform. To measure the propensity for users to switch between languages, we map languages as the nodes of a network, with weighted edges representing the number of users who posted in both languages on Koo. This layout follows the principle of a global language network (GLN), which allows us to quantify indirect communications between pairs of languages by looking at the number of speakers they share [70]. By considering the number of modal speakers in two linguistic communities and the number of speakers they share, we use the phi coefficient to measure the association between the two languages. For two languages *i* and *j*, their phi coefficient $\Phi_{ij}$ [70] is given by:

$$\Phi_{ij}=\frac{M_{ij}N-M_{i}M_{j}}{\sqrt{\left(M_{i}M_{j}(N-M_{i})(N-M_{j})\right)}},$$ (3)

with *N* being the total number of users, *M*_*i*_ and *M*_*j*_ being the number of speakers of language *i* and *j* respectively, and *M*_*ij*_ being the number of bilingual speakers for languages *i* and *j*. A positive phi coefficient indicates that the number of bilingual speakers between languages *i* and *j* is higher than what could be expected based on their representation in our dataset, whereas a negative value indicates that the co-occurrence of both languages is under-represented relative to the size of the communities. This metric therefore allows us to assess whether there is a stronger connection between two linguistic communities on Koo, than is expected due to chance alone.

To ensure that the link between two linguistic communities is significant, we use the *t* statistic, defined as:

$$t_{ij}=\frac{\Phi_{ij}\sqrt{D-2}}{\sqrt{1-\Phi_{ij}^{2}}},$$ (4)

where the degree of freedom *D* is defined as $D=\text{min}(M_{i},M_{j})$. This test is chosen to ensure consistency with existing analysis on global language networks [70]. As all the linguistic communities we consider in our analysis have at least 20 modal speakers, we set *D* = 20. By setting *p* = 0.05 (note that, due to the multiple comparison problem, we use FDR corrected p-values), we can reject the null hypothesis, i.e., the number of links between two languages in the global language network is not statistically significant, if $t_{ij}\geq 1.72$ (one-tailed *t*-test). Any significant link in the network indicates that there are significantly more bilingual speakers between two languages than expected by chance. Note, however, that we caution against over-interpretation of these results given that the *t*-test is underpowered when used with data which is heavy tailed (as is the case for our correlational data).

**Fig 5:**
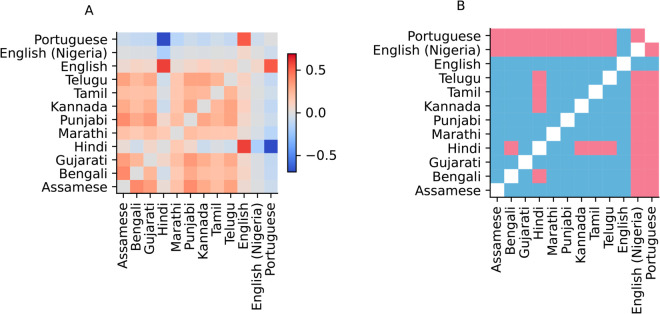
Global language network and multilingual activity. A) The correlation measured from the global language network. Two languages with a positive correlation share more connections than expected based on their respective number of speakers. B) The t-statistic for each pair of languages in the global language network. The t-statistic is applied to ensure alignment with the original global language network analysis in [70] – we note that correlational data is centrally distributed but non-normal, limiting the statistical power of the t-test; as such we recommend against over-interpretation of these results. Blue cells indicate that the link between the two languages is significant with respect to the t-statistic, whereas red cells highlight non-significant links. A link is considered significant if *p* < 0.05 (*t* > 1.72). Note that p-values have been adjusted using the Benjamini-Hochberg correction to mitigate false discoveries that arise due to the simultaneous testing of many different correlations, although this correction does not effect which language-pairs are found to by significantly correlated. Languages have been grouped according to language group. Indo-aryan: Assamese, Bengali, Gujarati, Hindi, Marathi and Punjabi. Dravidian: Kannada, Tamil and Telugu. Non-Indian: English, English (Nigeria) and Portuguese.

Fig 5A displays the value of the phi coefficient between the main languages used on Koo. Languages are ordered according to the language group to which they belong (Indo-aryan, Dravidian, or non-Indian languages). We notice that there is a positive association between the Indian languages used on Koo, particularly those belonging to the same language group. Despite being strongly homophilic, the Hindi-speaking community (Hindi is an Indo-aryan language) has a positive correlation with several Indian languages, all of which are also Indo-aryan languages including Gujarati and Marathi, but a non-significant correlation with the Dravidian languages (Telugu, Kannada, Tamil). Smaller linguistic communities in India also show a strong symbiosis in the network: languages such as Telugu, Kannada, Tamil and Assamese are strongly connected to one another, indicating that speakers of less prominent Indian languages on the platform are more likely to also communicate on Koo using another national language. Our findings are aligned with the results of the 2011 Indian language census, indicating a large number of bilingual and trilingual speakers among the smaller linguistic communities in the country [71]. On the other hand, both the Nigerian English and Portuguese-speaking communities have a negative correlation with respect to every other linguistic community, English excepted.

Fig 5B shows the results of the t-statistic, with significant links in the global language network highlighted in blue. We notice that both the Brazilian and Nigerian communities share non-significant links with the smaller linguistic communities on Koo, indicating that there is not a meaningful number of users who use both Portuguese or Nigerian English and another language on Koo - with the exception of English, which is significantly correlated to all languages. These results further suggest that the Brazilian and Nigerian communities were strongly isolated on Koo when it came to language mobility, whereas a shared cultural background enabled Indian language speakers to navigate through different linguistic communities on the platform.

### Discourse richness and similarity across languages

What about content? A natural question is whether stronger ties between two linguistic communities also implies that their respective discourses are similar. Moreover, some of the linguistic communities being larger than others, we hypothesize that a larger community should have a richer discourse. Studies have shown that the size of a community defines its propensity to sway the discussion topics in another community [72], and that nurturing a local discourse can allow a local community to claim their own governance [73].

To answer these questions, we use diversity measures from ecology, which were defined to assess the richness of an environment by looking at the presence of various species and their respective prevalence [74], as well as how often these species can be found across different environments [75]. These methods were also previously used in linguistics research, for example to measure linguistic diversity between Canadian cities [76]. For our analysis, we consider hashtags used within a linguistic community as a proxy for the discourse. Hashtags have been shown to occupy a different linguistic function than words, sharing similarities across languages [77], thus allowing us to capture narratives shared by various linguistic communities on Koo. Hashtags are also a more reliable signal to measure the overlap of narratives across linguistic communities than plain text, as hashtags can be identified without the need to compare textual data from different languages. Our approach is further motivated by the presence of several low-resource languages in our corpus, which are known to be under-represented in many large language models and can therefore lead to unreliable text classification [78].

To assess the richness of the discourse within a linguistic community, we use the alpha diversity, which measures the proclivity for an environment to host various species at a local scale. In our case, we compute the alpha diversity of hashtags used by a linguistic community using the Chao1 estimator. We can also estimate the propensity for two linguistic communities to discuss similar topics by computing the beta diversity, which assesses the propensity for the species composition of two environments to be similar. Using the hashtags, we measure the beta diversity of the discourse between two linguistic communities with the Bray-Curtis dissimilarity index.

To measure the richness of the discourse within a linguistic community, we compute the alpha diversity with the Chao1 estimator ${\hat{S}}_{Chao1}$, which aims at providing a lower-bound estimation of the number of unseen species, in order to assess the total number of unique hashtags used by a linguistic community from our observations. The Chao1 estimator is defined as:

$${\hat{S}}_{Chao1}=\left\{ \begin{array}{cc} S+\frac{N-1}{N}\frac{s_{1}^{2}}{2s_{2}} & s_{2}>0\\ S+\frac{N-1}{N}\frac{s_{1}(s_{1}-1)}{2} & s_{2}=0 \end{array}\right.,$$ (5)

where *S* is the total number of hashtags, *N* is the number of unique hashtags and *s*_*i*_ is the number of hashtags that appear at least *i* times in the observations. This formula, however, only takes into account hashtags that appear once or twice in a linguistic community. To extend the amount of information used to estimate the richness of the conversation, we compute the improved Chao1 estimator ${\hat{S}}_{iChao1}$, which aims at correcting the first-order bias of ${\hat{S}}_{Chao1}$ by also including the number of triplets *s*_3_ and quadruplets *s*_4_. The improved Chao1 estimator is defined as [79]:

$${\hat{S}}_{iChao1}={\hat{S}}_{Chao1}+\frac{N-3}{4N}\frac{s_{3}}{s_{4}}\cdot max(s_{1}-\frac{N-3}{N-1}\frac{s_{2}s_{3}}{2s_{4}},0).$$ (6)

**Fig 6:**
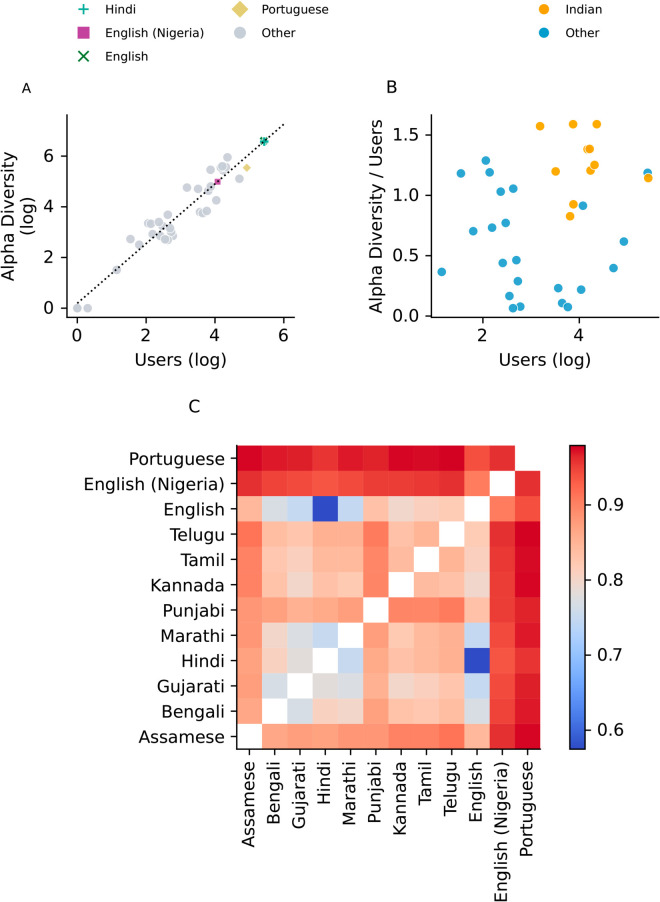
Discourse richness and similarity across linguistic communities on Koo. A) The alpha diversity of the discourse in a linguistic community, measured with the improved Chao1 estimator, plotted against the population size of the community, along with the linear fit (Spearman’s *R*^2^ = 0.92). Colours are used to indicate the main linguistic communities on Koo. B) The ratio between the improved Chao1 estimator and the population size plotted against the population size of the community. Colours indicate communities speaking an Indian language. C) The beta dissimilarity, measured with the Bray-Curtis index by considering the list of hashtags used by the largest linguistic communities on Koo and measuring their respective dissimilarity. Two communities with an index close to 0 use similar hashtags, whereas an index of 1 indicates that there is no overlap in the hashtags used by the communities. Languages have been grouped according to language group. Indo-aryan: Assamese, Bengali, Gujarati, Hindi, Marathi and Punjabi. Dravidian: Kannada, Tamil and Telugu. Non-Indian: English, English (Nigeria) and Portuguese.

Fig 6A shows the improved Chao1 estimator for each linguistic community plotted against its population size. We measure a strong correlation (*R*^2^ = 0.92) between the two variables, with bigger communities displaying a richer use of hashtags than smaller ones. There is, however, a non-negligible fluctuation in the measured richness for linguistic communities that share a similar population size, especially when looking at mid-sized communities.

To look more closely at this disparity, we display on Fig 6B the improved Chao1 estimator per user, plotted against the population size. Points are coloured to highlight the Indian official languages. Interestingly, we notice that for a similar population size, the ratio is higher for Indian languages than for non-Indian languages (with English, a language widely used in Indian administration, being the exception).

These findings suggest that, with similar population sizes, Indian language speakers gave rise to richer discourses on Koo than non-Indian linguistic communities. These findings are especially insightful when considering that the Portuguese and Nigerian English speaking users were involved in far less rich conversations than their Indian peers, demonstrating how Koo was unable to build a sustained user base outside its native India [47].

The presence of a rich discourse among Indian communities does not necessarily indicate the existence of national cohesion. In order to identify cultural divides in the use of language, we can measure the Bray-Curtis dissimilarity index [80], a measure of beta diversity, defined as

$$\beta_{AB}=\frac{\sum_{i}|p_{i}^{A}-p_{i}^{B}|}{\sum_{i}(p_{i}^{A}-p_{i}^{B})},$$ (7)

where $p_{i}^{A}$ and $p_{i}^{B}$ represent the relative frequency of hashtag *i* in the linguistic communities A and B, respectively. $\beta_{AB}$ provides us with a measure of the dissimilarity between the two environments: if the two linguistic communities use no similar hashtags, their dissimilarity $\beta_{AB}$ will be 1, whereas it will be equal to 0 if they use exactly the same hashtags.

Fig 6C shows the Bray-Curtis dissimilarity between the discourse in two linguistic communities. We notice that the Portuguese and Nigerian communities have a high dissimilarity when compared to the other languages, which indicates that there is a low overlap in the hashtags shared by Portuguese and Nigerian English speakers with respect to other linguistic communities. This finding further confirms their status as isolated clusters.

Looking at the Indian languages, we also notice a higher similarity shared between a few national languages, namely Gujarati, Bengali, Hindi and Marathi. These four languages are Indo-Aryan languages, thus sharing more linguistic similarities with each other than with Dravidian languages such as Telugu and Kannada. These results therefore outline the existence of a *Sprachbund*, a set of languages that share many similarities in their structure [81]. This concept seeps within the online discourse on Koo, where we see a strong affinity between languages that share similar roots. Overall, Indian languages share a more similar discourse with each other than with non-Indian languages on the platform, which might explain why the conversation is richer amongst these communities. Future research should consider further investigating whether greater similarity in online discourses can improve user retention amongst smaller linguistic communities.

## Discussion and conclusion

In this paper, we have analyzed the emergence of a multi-lingual ecosystem on Koo, a micro-blogging platform which was founded and based in India, shaped by successive migrations prompted by political and social events in India, Nigeria, and Brazil. This study follows our previous work on Koo where we discussed these migrations and released the Koo dataset [15]. In the current paper, we first looked at the impact of collective migrations to Koo in India, Nigeria and Brazil, and measured their respective success by comparing their impact on the daily registrations on Koo and their user retention over time. Second, we looked at the user interaction network and showed a strong linguistic segregation within isolated communities and the propensity for Hindi- and English-speaking users to be more central in the overall network. Third, we measured the average commitment and homophily for each linguistic community, and showed that linguistic communities with larger user-bases are more likely to be self-sufficient and dominantly display in-group interactions, with English being the exception due to its role as a bridging language across cultural communities, and its extensive use in Indian politics and society. Fourth, we generated the global language network and highlighted the importance of linguistic crossovers between Indian languages, notably within the Indo-aryan language family, and within the Dravidian language family. In contrast, Portuguese-speaking users only significantly overlapped with English speaking users. Finally, we measured the richness and dissimilarity of linguistic clusters and noticed a strong interconnection across Indian languages, which also had a richer discourse than other communities with similar population sizes. These results could be interpreted as suggesting that richer cultural discourses increase user retention (future work is needed to fully confirm whether this is true, in general), making platforms more sustainable in the long term. However, our results emphasize that despite the many different languages used on Koo, most linguistic communities remained relatively small when compared to uptake in English or Hindi. This may have played a part in Koo’s failure to remain sustainable in the long term following an improvement in relations between the Indian government and Twitter after hostility in 2020, and especially following Twitter’s change of ownership and rebranding to X.

Our study offers a first insight into the emergence, growth, and ultimate failure of a multi-lingual alt-tech platform, where the co-existence of diverse linguistic communities was driven by independent collective migrations. Despite Koo’s ambitions to unite the non-English speaking world under a single banner, the language divide we measure both in terms of interactions and discourse similarity suggests that these communities grew concurrently on the platform but without a strong tendency to overlap. While major languages can still thrive and be used by a sustainable community, less prevalent languages end up being marginally used, leading their speakers to be less involved in the major conversations taking place on the platform. These findings might indicate that minority linguistic communities are disenfranchised from the central political discourse on social media. Previous inquiries have already raised concerns about the access to many public services in India for local language speakers [82].

Our analysis highlights the growth of the digital market in the Global South, which is often overlooked by the academic literature. Koo pledged to offer an online space catering to non-English speaking users and managed to attract key political figures from major emerging economies, leading the platform to become a serious competitor to Twitter [83] for a period of time. Moreover, our research adds more nuance to the literature on alternative platforms that has been mostly focused on right-wing narratives. Koo was used by figures from both the Indian and the Brazilian governments, representing contrasting political orientations. However, the Indian and Brazilian communities on Koo were shown to hardly interact with one another, indicating scarce engagements across the political spectrum. Koo’s struggles to attract members of political parties other than the BJP and their allies in India highlights the difficulties alt-tech platforms face in building an online space for politically diverse communities [84]. This is further exacerbated by the recent rise of policies related to digital sovereignty across a number of countries, compromising Koo’s ambition to become a unifying platform for communities outside the West [85].

Our findings are limited by several factors, which can be explored in future studies. First, our analysis is restricted to Koo, which was the most popular micro-blogging platform based in India for a period of time, and the second most popular India-based social platform in general (after ShareChat). Social platforms based in other nations in the Global South may display different structural patterns and should also be studied. Future studies may consider extending our analysis to other popular platforms based in India including ShareChat, 2go and Line, and should consider analyzing platforms comparatively to ensure that platform-specific results can be understood in relation to the behavior observed on competing platforms (see for example [86,87]). This extension would allow us to study, for example, whether Koo’s multi-language support fostered rich linguistic communities beyond what is observed on other platforms (such as Twitter, now X) where these languages can be used even if not explicitly supported by the platform. Unfortunately, this comparative research is reliant on appropriate data availability which has proved difficult since multiple social media platforms have restricted academic data access. Second, our analysis relies on the language identified by Koo’s automated system for each post and comment on Koo. This may be problematic given that automatic language detection has various levels of reliability depending on the source language [88]. Further work may consider the accuracy of Koo’s language identification pipeline, and whether it results in specific biases in our analysis. Finally, our work focuses on a static overview of Koo’s interaction network by looking at all the user activity that took place over the four years studied. This ensures that we are able to fairly compare diverse linguistic communities who joined Koo at different times. However, an analysis of the temporal evolution of communities on Koo would be equally valuable, in particular to identify how the network evolved following each collective migration.

Overall, our study improves our understanding of social interaction patterns within multilingual communities by looking beyond western social platforms. We anticipate that our work will inspire more research on the global social media ecosystem, to ensure that our findings with respect to alternative platforms are nuanced by cultural and linguistic factors. Finally, our analysis stresses the need to develop a wider range of tools to analyze social media content in minority languages, many of which are currently under-represented on the internet, and understudied by academics.

## Supporting information

S1 File Supplementary information document. PDF file containing all supporting information including 5 supplementary figures.
(PDF)
