# Supplementary material for: How language, culture, and geography shape online dialogue: Insights from Koo
Source: PLoS One. 2025 Aug 21;20(8):e0329838. doi: 10.1371/journal.pone.0329838 (PMC12370020; doi:10.1371/journal.pone.0329838)
Supplement: S1 File — PDF file containing all supporting information including 5 supplementary figures. (PDF) [file pone.0329838.s001.pdf]

Supplementary File 1.
How Language, Culture, and Geography shape Online Dialogue: Insights from Koo

Amin Mekacher[1], Max Falkenberg[2] and Andrea Baronchelli[1,*]

[1] *City University of London, Department of Mathematics, London EC1V 0HB, (UK)*
[2] *Central European University, Department of Network & Data Science, Vienna 1120, (AT)*
[*]Corresponding author: andrea.baronchelli.1@city.ac.uk
(Dated: July 24, 2025)

A. Linguistic communities' longitudinal evolution

We showed previously that Koo experienced several growth spurs, following collective migrations from various demographics. We show that these additional registrations also led to an increase of activity coming from these groups, by breaking down the daily activity by linguistic communities. Figure 1 displays the moving average of daily registrations for each linguistic community. As we can see, the peaks that we noticed previously are indeed caused by the BJP migration, the Nigerian ban on Twitter and the Brazilian community moving to Koo. Interestingly, each peak also leads to a moderate growth of interest among other linguistic communities.

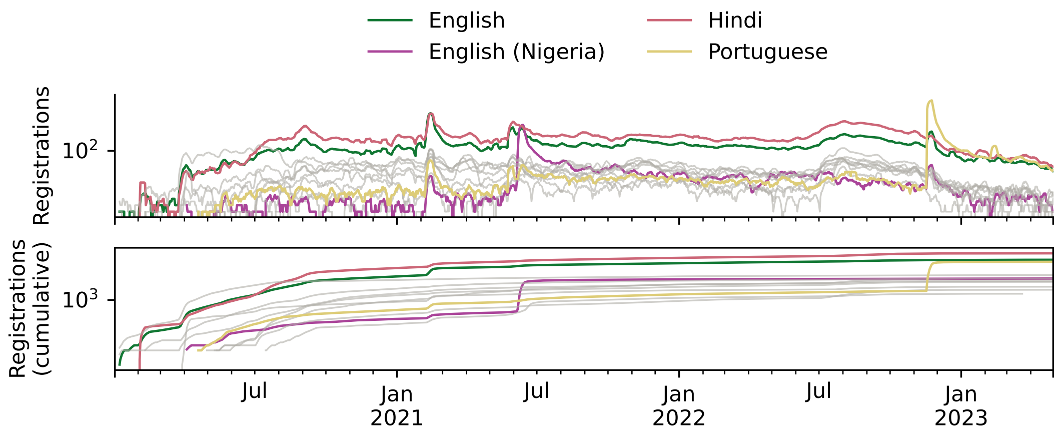

FIG. 1. **Registration activity from linguistic communities.** Time-series showing the 7-day moving average of the number of registrations made by each linguistic community. The major communities are highlighted in color.

Figures 2, 3 and 4 display the moving average for the number of comments, shares and likes made by each linguistic community on a daily basis, showcasing a burst of activity on days related to the collective migrations observed on Koo.

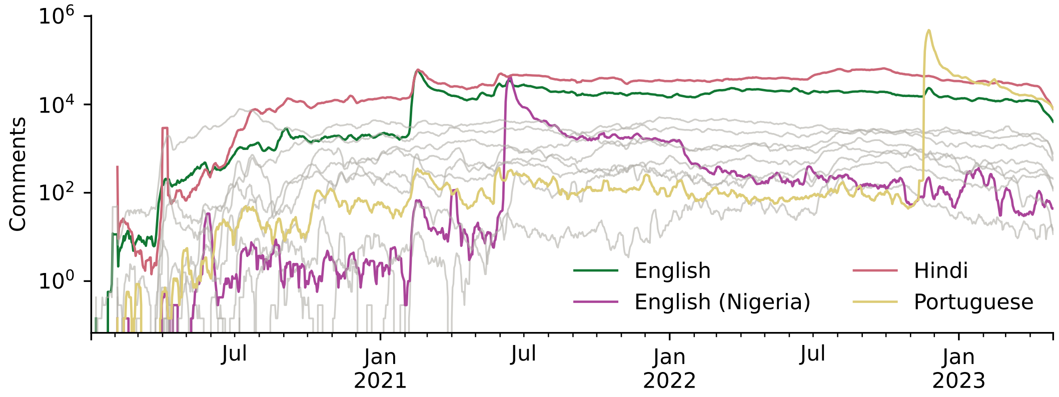

FIG. 2. **Commenting activity from linguistic communities.** Time-series showing the 7-day moving average of the number of comments made by each linguistic community. The major communities are highlighted in color.

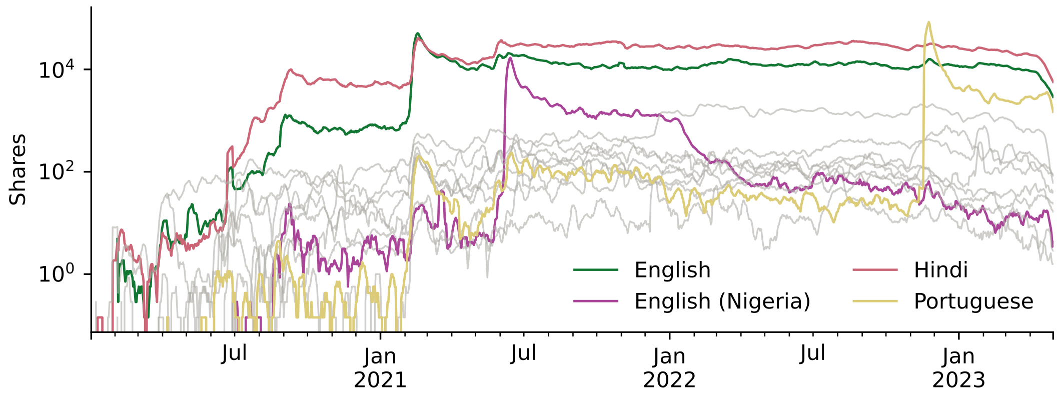

FIG. 3. **Sharing activity from linguistic communities.** Time-series showing the 7-day moving average of the number of shares made by each linguistic community. The major communities are highlighted in color.

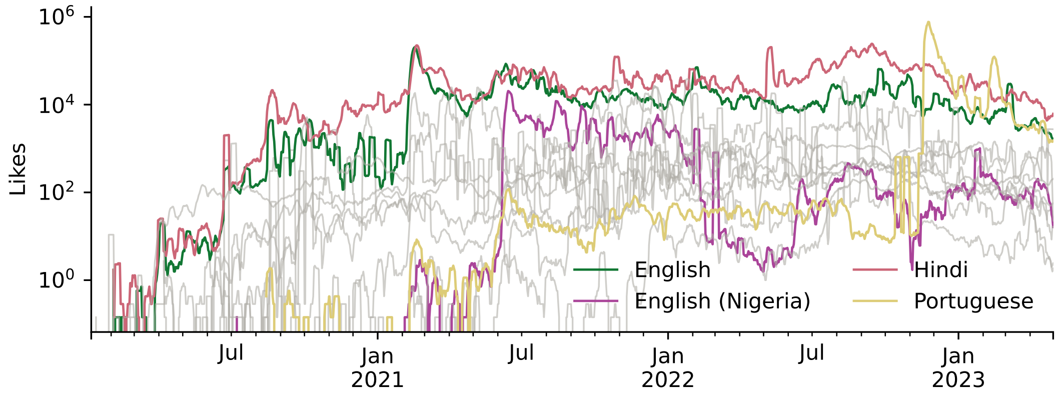

FIG. 4. **Liking activity from linguistic communities.** Time-series showing the 7-day moving average of the number of likes made by each linguistic community. The major communities are highlighted in color.

B. Interaction network k-core analysis

Our analysis highlighted a strong dominance of English- and Hindi-speaking users on the k-core of the interaction network on Koo. Figure 5 displays the distribution of users in the k-core of the network, for each linguistic community. The inset zooms on the highest values of k observed in the network, to further underline that only English and Hindi speakers are included in the highest cores.

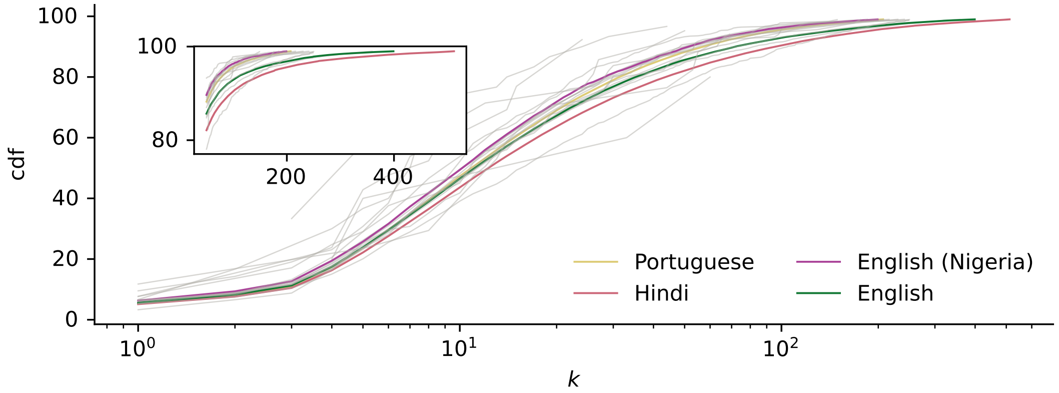

FIG. 5. **Linguistic composition of the k-core.** Cumulative distribution function of the percentage of users from a given linguistic community belonging to the k-core, for every value of k. Inset: Zoom on the distribution for $k \geq 50$
